# Suicide: A Modern Obsession

**DOI:** 10.1192/pb.bp.116.053645

**Published:** 2016-12

**Authors:** Teifion Davies

If the poor have been with us always, then so too has suicide. I read Al Alvarez's *The Savage God* as a preclinical student trying to make sense of the sudden and unexplained death of my beloved uncle. His death, so many years ago, still rends my family in two: those who sympathise with and those who accuse his widow. Alvarez, who was himself trying to untangle the suicide of his friend the poet Sylvia Plath and the emotions generated by it, provided some valuable insights but precious little comfort. In many cultures it had always been so, it seemed, with all associated with such an act – whether dead or surviving, aware or unaware of the victim's intentions – being held blameworthy for commission of sin or omission of care by some in the victim's family, community, religion or state.

The search for answers to the murky and emotionally laden questions surrounding suicide is widespread. This book – written by a psychiatrist and a journalist – attempts to answer many of the frequently asked questions and poses some others to ‘tease out fact from fiction, pragmatism from hysteria and common sense from nonsense’ (p.16). The book is biased towards general-interest readers and arose from the authors' concerns at the proliferation of suicide prevention programmes in a country (Ireland) with a relatively low suicide rate – the modern obsession referred to in the title.The structure and style have elements of investigative journalism rather than either patient support manual or academic treatise, with headings such as ‘Why?’ (p. 38) and ‘Irresponsible and responsible media reporting of suicide’ (p.130). The content is composed chiefly of a fairly conventional but selective review of the topic of suicide, sprinkled with case studies and extracts from interviews with experts conducted by one of the authors.

Had this book, rather than Alvarez's, been available to me, would it have provided the answers and comfort I sought? Sadly, no, it would have left me frustrated by its rather self-defensive tone and by statements of what might be obvious to a seasoned mental health professional but not to a general-interest reader nor a grieving relative. All those well-known risk factors (with such feeble positive predictive value) and ‘Safety-planning interventions’ (p.186) militate against accepting that ‘Not all suicides are preventable and there are no foolproof measures … ’ (p. 214). And no amount of understanding could expiate the guilt.

